# A comparative analysis of physical fitness characteristics and rugby-specific game skills of schoolboy players by playing standards: Implications for talent identification programs in resource-constrained settings

**DOI:** 10.17159/2078-516X/2024/v36i1a18525

**Published:** 2024-01-15

**Authors:** M Chiwaridzo, T Mudawarima, TW Shumba, GD Ferguson, BC Smits-Engelsman

**Affiliations:** 1 Department of Occupational and Physiotherapy, Faculty of Health Sciences and Veterinary Medicine, University of Namibia, Windhoek, Namibia; 2 Rehabilitation Unit, Department of Primary Health Care Sciences, Faculty of Medicine and Health Sciences, University of Zimbabwe, Harare, Zimbabwe; 3 Department of Physiotherapy, DDT College of Medicine, Gaborone, Botswana; 4 Division of Physiotherapy, School of Health and Rehabilitation Sciences, University of Cape Town, Cape Town, South Africa

**Keywords:** rugby, physical characteristics, adolescents, youth

## Abstract

**Background:**

Rugby has not grown extensively in Africa compared to other continents, necessitating talent identification (TID) programs to recruit junior talent. However, it is unclear which physical characteristics and rugby-specific game skills to base the objective recruitment of potentially talented young players.

**Objectives:**

This study profiled the physical fitness characteristics and rugby-specific game skills of schoolboy rugby players by playing standards to identify variables differentiating elite from sub-elite players from Under 16 (U16) to U19 age categories. The study further compared Zimbabwean cohort data with similar data from international/regional countries.

**Methods:**

A cross-sectional design was utilised with 158 Zimbabwean schoolboy players playing competitive (elite) and noncompetitive (sub-elite) rugby. The participants were measured for anthropometrics, speed, agility, upper-and-lower-muscular strength/power, muscle flexibility, prolonged high-intensity intermittent running ability, repeated high-intensity exercise performance ability, tackling, passing, and catching abilities.

**Results:**

For U16s, Vertical Jump (VJ), 2kg Medicine Ball Chest Throw (2kg MBCT), Wall-Sit-Leg Strength (WSLS), Yo-Yo Intermittent Recovery Level 1 (Yo-Yo IRL1), Tackling and Catching Ability tests discriminated elite from sub-elite players. Among U19s, the following tests differentiated elite from sub-elite players: VJ, 2kg MBCT, WSLS, 20-m/40-m linear speed, 60-s Push Up, One-Repetition Maximum Back Squat (IRM BS), 1RM Bench Press (BP), Repeated High-Intensity Exercise (RHIE), Tackling and Passing Ability. Elite Zimbabwean schoolboy rugby players were significantly leaner, slower, and “weaker” than their international/regional counterparts.

**Conclusion:**

The results suggest important physical fitness characteristics and rugby-specific game skills for future identification of potentially talented players at U16/U19 categories.

Approximately 10 million people play rugby union (RU) at different playing standards worldwide.^[1]^ However, over three-quarters of global elite players originate from developed countries, including England, Australia, France, and New Zealand (NZ).^[2]^ Despite efforts from World Rugby (WR) to spearhead RU development globally, RU in Africa lags in many fundamental aspects critical for its popularity and growth, such as grassroots development and talent identification (TID).^[3]^ This is concerning and necessitates efforts from relevant stakeholders, including researchers, to provide data capable of informing TID programs. Such programs may improve rugby expansion in Africa by mass identifying junior players likely to excel in this sport.^[4]^

Implementing rugby-specific TID programs is a complicated process requiring consideration of important factors that influence immediate success and long-term sustainability.^[4]^ Invariably, the process commences with conceptualising a talent search strategy leading to the actual implementation. Often, TID initiatives in Africa struggle with take-off because of a lack of substantial financial investment.^[5]^ If they succeed in being launched, they are further hampered by a lack of scientific basis underpinning the formulation and implementation.^[6]^ In Zimbabwe, for example, anecdotal evidence has shown that TID initiatives are usually led by a pre-selected group of recruiter coaches whose recruitment strategies are unclear. Understandably, the coaches base talent search on their previous playing experience and pre-conceived notion of ideal players. Although that strategy allows for the convenient recruitment of many potential players, the approach lacks objectivity. It is susceptible to omitting players with greater talent that may only show entirely in the future. ^[7]^ Notwithstanding the importance of coaches’ subjective experience, the TID recruitment programs should be objective and guided by the rugby-specific game skills or physical characteristics known to discriminate skilled from less-skilled rugby players.^[8]^ That simplistic approach standardises junior talent searches by the recruiter coaches. Moreover, it provides an objective framework for packaging TID programs rather than only relying on the coaches’ experience.

Rugby-specific game skills and physical attributes are known to differentiate rugby players by playing standards.^[9]^

However, it is unclear which specific physical fitness characteristics and rugby-specific game skills recruiters should focus on to recruit potential young players during planned TID initiatives. This is especially so for developing African countries such as Zimbabwe where rugby development is still embryonic.^[8]^ RU is a sport characterised by highly demanding and repetitive episodes of physical exertion.^[2]^ Therefore, one plausible strategy to establish the physical fitness characteristics and rugby-specific game skills relevant for incorporation in objective TID programs is to identify qualities which significantly differentiate junior rugby players by playing standards. Such studies are limited to Africa, but they are an important source of information highlighting the possible differences in player characteristics between junior RU players playing at different competitive levels. Additionally, limited studies compare schoolboy RU players from African countries hardly known at the international level, such as Zimbabwe, with similar players playing at the highest competitive level from countries such as England, France, and NZ.

In a cross-sectional study comparing junior rugby players playing at different playing standards, Spamer et al.^[10]^ found that elite Under 16 (U16) NZ schoolboys, representing higher-level players, outperformed elite U16 South African (SA) schoolboys for body mass, upper-body muscular endurance, lower-body muscular power, flexibility, 10-m speed, agility, air-and-ground skills, and passing-for-distance. These findings provide a basis for identifying essential skills and characteristics to be included in a TID program in SA. In England, Dimundo et al.^[11]^ found that talent-identified U15 RU players had greater body mass and handgrip strength scores than non-selected players. Such evidence can be amalgamated to develop rugby-specific TID programs incorporating routine assessments of body mass and strength.^[11]^ However, this existing evidence is mainly from developed countries with exceptional standards for international rugby. It cannot be directly extrapolated to other settings, such as Zimbabwe, which is hardly known for playing competitive rugby.

Therefore, this study aimed to profile Zimbabwean schoolboy rugby players’ physical fitness characteristics and rugby-specific game skills by playing standards. This enabled the identification of the variables significantly capable of differentiating elite from sub-elite schoolboy rugby players from the U16 to U19 age categories. Furthermore, the study compared data for physical fitness characteristics and rugby-specific game skills obtained from the Zimbabwean cohort of U16 and U19 players with similar data from junior players obtained from England, France, NZ, and SA, countries representing a higher level of competition. The study’s significance lies in providing an objective framework for identifying future rugby talent based on the differences in physical fitness characteristics and rugby-specific game skills between elite and sub-elite junior players in Zimbabwe and outside. These study results will likely facilitate the expansion and professionalism of rugby in Zimbabwe through objective schoolboy player recruitment strategies.

## Methods

### Research setting and participants

The study targeted schoolboys playing competitive and noncompetitive rugby in secondary schools based in Harare, Zimbabwe. Schoolboy rugby is the only organised pathway for junior rugby participation in Zimbabwe. At the time of the study, two schoolboy rugby leagues defined rugby playing standards in the country, namely the Super Eight Schools Rugby League (SESRL) and Co-Educational Schools Rugby League (CESRL).^[8]^ The former is the highly competitive league (elite), featuring only eight schools. The SESRL players are exposed to superior coaching services, health care, scholarships, corporate-sponsored training gear and facilities, and opportunities for regional/international tours and competitions. In contrast, the CESRL is the second most competitive league (sub-elite) also composed of eight schools.

An a priori calculation required a minimum sample size of 120 participants to detect a possibly large mean difference of 300m in the Yo-Yo Intermittent Recovery Level 1 (Yo-Yo IR LI) test scores between skilled and less skilled groups. This was based on Jones et al.^[12]^ findings with the type I error set at 0.05 and 80% power. During the study, there were 165 players playing as U16s and U19s in four schools based in Harare playing SESRL. One school was purposefully selected based on the fact the school had won the SESRL thrice in the last five competitive seasons. All the boys playing in the U16 and U19 first teams were invited to participate. From the CESRL, one school was randomly selected to provide a sample of U16s and U19s representing the “sub-elite” players.

### Eligibility criteria

Only schoolboy rugby players who assented after receiving their parents’/guardians’ consent were included in the study. Participants had to be fit to partake in physical activities based on the Physical Activity Readiness Questionnaire (PAR-Q).^[13]^ Participants with self-reported medical conditions contraindicating physical activities were excluded. Furthermore, parents/caregivers had to corroborate supplied medical information by completing an Adolescent Medical Health Questionnaire (AMH-Q) eliciting participants’ medical history.

### Outcome measures

Participants completed a questionnaire soliciting demographic and sport-related information. The coaches corroborated all the information. The test battery (Supplementary file) measured physical fitness and rugby-specific game skills. The development of the test battery has been explained elsewhere.^[8]^ Briefly, the test battery assessed anthropometrics, speed, agility, upper-and lower-muscular strength/power, muscle flexibility, prolonged high-intensity intermittent running ability, repeated high-intensity exercise performance ability, tackling proficiency, passing, and catching ability. Only U19 rugby players performed one-repetition maximum bench press (1RM BP) and back squat (1RM BS) tests because of allowed exposure to resistance training. The U16s performed the 60-s Push-Up and Wall Sit Leg Strength (WSLS) tests instead of 1RM BP and 1RM BS, respectively.

### Procedures

Ethical approval was granted by the Medical Research Council of Zimbabwe (MRCZ ref: A/2070). Written informed consent and assent were obtained from parents and players, respectively. Elite U16 and U19 participants were measured in a preliminary test-retest study to estimate the relative reliability of each test item.^[14]^ Before the main study, all participants were familiarised with the test battery on two consecutive days. The main study testing occurred during training during the competitive rugby season. Participants continued their normal diet, school class schedule, and training programs. They were asked to refrain from ingesting caffeine and performance enhancers.

### Statistical analysis

T-tests were used to compare the normally distributed data and Mann–Whitney U tests were used for non-parametric analysis. All statistical analysis was conducted using Statistica version 13.2 (Statistica, Tulsa, USA).

## Results

Table 1 depicts results for physical characteristics and rugby-specific game skills of 158 schoolboy U16s and U19s by playing standards. For each age category, participants were similar in chronological age, playing experience, and skinfold scores regardless of playing standards. Among U16s, the Vertical Jump (VJ), 2kg Medicine Ball Chest Throw (2kg MBCT), Wall Sit Leg Strength (WSLS), and the Yo-Yo Intermittent Recovery Level 1 (Yo-Yo IRL1) tests significantly differentiated elite from the sub-elite players. The elite players showed better variable scores (p<0.05) for explosive lower-leg muscular power, upper-limb muscular power, lower-leg isometric muscular strength and prolonged high-intensity intermittent running ability. Additionally, elite players showed better tackling and catching ability test scores (p<0.01). Among U19s, the following tests differentiated elite from the sub-elite players, with the elite players showing superior scores (p<0.05) for the VJ, 2kg MBCT, WSLS, 20-m and 40-m linear speed, 60-s Push Up, IRM BS, 1RM BP, Repeated High-Intensity Exercise (RHIE), Tackling and Passing ability.

Table 2 depicts a comparative analysis of data on physical characteristics and rugby-specific games skills between a Zimbabwean cohort of schoolboy rugby players with data derived from selected published studies conducted in England, France, NZ, and SA.^[12, 16–20]^ For U16s, largest practical significant differences were found for body mass (ES=2.2), 10m linear speed test (ES=4.0), 20m linear speed test (ES=2.0), 40m linear speed (ES=1.7), VJ test (ES=2.3) with international/regional elite U16 players outperforming the Zimbabwean cohort. However, the Sit-and-Reach flexibility scores for Zimbabwean players (6.1±5.1cm) were significantly better compared to reported scores for elite SA U16 players (2.4±2.3cm) and NZ U16 players (−2.2±8.8cm).^[10]^ Additionally, moderate practical differences were found for the Yo-Yo IR L1 test (ES=0.6) with the compared to reported scores for an English cohort Zimbabwean U16 cohort achieving higher values (1307±229m) compared to reported scores for an English cohort (1145±337m).^[16]^ Collectively, these results indicate that elite Zimbabwean U16 rugby players are significantly leaner, slower, “weaker”, more flexible and had more endurance than their international/regional counterparts. Trivial differences were found for height (ES=0.0), sum of skinfolds (ES=0.2) and 60s Push Up (ES=0.0) test scores between the Zimbabwean players and the international/regional players.

For U19s (Table 3), large practical significant values were found for body mass (ES=3.2), 10m, 20m, 40m linear speed tests (ES=1.8–4.3) and 1RM BP (ES=1.0) test with international/regional players showing significantly better scores compared to Zimbabweans. However, for the Yo-Yo IR L1 test, the Zimbabwean U19 cohort achieved significantly higher values (1506±76m) compared to previously reported scores for an English cohort (1022±515m) with an ES of 1.3.^[12]^ Trivial practical differences were found for height (ES=0.0) and sum of skinfolds (ES=0.2) test scores between the U19 Zimbabwean cohort and the international/regional players.

## Discussion

This study analysed the physical fitness characteristics and rugby-specific game skills of Zimbabwean schoolboy rugby players to identify variables differentiating elite from sub-elite rugby players. The information was further compared with similar data from international/regional studies. The rationale for comparing this data was to build an objective framework for identifying future talent based on the differences in physical fitness characteristics and game-specific skills between *(1)* elite and sub-elite schoolboy players in Zimbabwe and *(2)* between elite Zimbabwean players and international/regional players. Although fraught with limitations, such studies highlighting the magnitude of cross-sectional differences in physical and skill qualities between players of different playing standards are essential. They establish tentative baseline values useful for participant evaluations in TID recruitment programs conducted in resource-constrained settings.

One key finding was that U16 Zimbabwean elite players had better scores for upper-and lower-limb muscular strength/power, prolonged high-intensity intermittent running ability, tackling and catching ability compared to the sub-elite players. These findings confirm the shared notion in literature that schoolboy rugby players should be strong, fit, and exhibit exceptional skills such as tackling and catching. ^[12, 19]^ Generally, the present study findings suggest to rugby coaches the importance of objectively profiling both physical characteristics and rugby-specific game skills of potential players when recruiting future rugby talent from the U16 level. This suggestion is logical considering that RU is characterised by highly repetitive episodes of physical exertion, necessitating the utilisation of upper and lower extremities in locomotor and technical activities such as sprinting, tackling, and catching. ^[2]^ Specifically, Zimbabwean coaches should aim to objectively recruit, from the available pool of potential junior talent, U16players with well-developed upper- or lower-body catching ability skills informed by the referenced values established in the present study. The obtained test scores for these qualities can be regarded as the baseline values for muscular strength/power, endurance, tackling proficiency, and consideration during TID programs for U16 players.

Thereafter, longitudinal training and development of the identified players should aim towards the qualities international/regional players have that are better than local players, such as appropriate body mass, speed, and explosive lower-leg muscular power. With sufficient specialised and focused training, the scores obtained for elite international/regional players should help set the maximum values for possible attainment by the elite Zimbabwean cohort.

For U19 players, the present study found that elite players outperformed sub-elite players with regards to the following tests: VJ, 2kg MBCT, WSLS, 20m and 40m linear speed, 60-s Push Up, IRM BS, 1RM BP, RHIE, Tackling and Passing ability test. Collectively, these results suggest the importance of assessing linear sprinting ability, upper-and-lower body muscular strength/power, repeated high-intensity performance ability, and tackling and passing abilities in potential U19 rugby talent. Practically, these findings highlight to coaches the physical characteristics and skills discriminating elite from subelite players and the attributes important for selection consideration. Given the large effect sizes observed for body mass, linear speed, and 1RM BP in favour of international/regional players, emphasis should be placed towards specialised conditional training. This elevates the qualities and skills of elite Zimbabwean players to levels aligned with international/regional counterparts. Cognisant of the influence of environmental and genetic factors, it is also possible that current strategies used to train and develop general endurance for the schoolboy players are probably being successful as the Zimbabwean U19 cohort achieved significantly higher values compared to previously reported scores for an English cohort.^[12]^

### Study strengths and limitations

This study uniquely analysed Zimbabwean schoolboy rugby players’ physical fitness characteristics and rugby-specific game skills by playing standards using validated tests. The present study findings were further compared to international/regional studies to suggest physical qualities and skills important for TID programs, player recruitment, and longitudinal training. This is the first study in Zimbabwe that has practical implications for TID initiatives. However, the findings should be interpreted while being cognisant of certain limitations. The cross-sectional design of the study precludes definite generalisations on the association between identified physical characteristics, rugby-specific game skills and future rugby success. Repeat studies and robust longitudinal study designs are needed to further ascertain the connection between these qualities and rugby success. This study only focused on physical fitness characteristics and rugby-specific game skills capable of differentiating elite from sub-elite rugby players. However, other factors such as psychological qualities, nutrition, biological maturation, training differences among others may need consideration for the development of successful holistic TID initiatives in Zimbabwe. The study compared data for physical fitness characteristics and rugby-specific game skills obtained from the Zimbabwean cohort of U16 and U19 players with similar data of male junior players obtained from England, France, NZ, and SA. Although these countries represent a higher playing standard, country selection was arbitrary. International/regional studies utilising a test battery similar to the present study’s test battery were non-existent. This made it difficult to directly compare with other studies for each test item in the test battery. Hence, test items, especially for the rugby-specific game skills, were not compared because of differences in test procedures between studies. Additionally, important factors such as positional differences of players, training volume, load, frequency, rugby training style, strength/power conditioning strategies were difficult to control between the included studies

## Conclusion

The physical and skill qualities of schoolboy RU players should be a key consideration for coaches during TID. This study showed that U16 Zimbabwean elite players had significantly better scores for upper-and lower-limb muscular strength/power, prolonged high-intensity intermittent running ability, tackling and catching ability compared to sub-elite players. The results suggest important qualities to aim for the future identification of schoolboy RU players. For the senior adolescent players, coaches should be aware that linear sprinting ability, upper-and lower body muscular strength/power, repeated high-intensity performance ability, tackling and passing abilities differentiate elite from sub-elite players. Therefore, the results of this study provide data for this unique population of Zimbabwean schoolboy rugby players. Elite Zimbabwean rugby players, regardless of age category, were significantly leaner, slower, and weaker than their international/regional counterparts. Practically, Zimbabwe adolescent coaches can set specific training goals to improve the baseline measures of talent-identified players to maximum values set by the scores recorded for international/regional elite players.

## Supplementary Information



## Figures and Tables

**Table 1:** Anthropometric, physical fitness characteristics and rugby-specific game skills of elite (n=41) and sub-elite (n=30) male adolescent players (Mean±SD)

	U16			U19		

Variables	Elite (n=41)	Sub-Elite (n=30)	Statistics t(df=69), p	Elite (n=41)	Sub-elite (n=46)	Statistics t(df=85), p

Chronological age (y)	14.9±0.3	14.8±0.4	1.1, 0.3	17.5±0.6	17.4±0.9	0.5, 0.6
Playing experience (y)	2.5±0.5	2.2±0.7	1.8, 0.1	5.0±0.7	4.9±0.7	0.4, 0.7

**Anthropometrics**						

Body mass (kg)	63.7±9.1	61.2±15.5	0.9, 0.4	77.5±9.6	75.9±11.6	0.7, 0.5
Height (m)	1.7±0.1	1.7±0.1	−0.5, 0.6	1.7±0.1	1.7±0.1	0.7, 0.5
Biceps (mm)	5.6±1.7	6.6±1.1	−2.4, 0.0	6.7±3.6	6.6±3.1	0.2, 0.9
Triceps (mm)	9.9±3.3	9.9±1.9	−0.01, 1.0	9.4±3.0	9.8±4.6	−0.5, 0.6
Subscapular (mm)	10.9±2.7	11.3± 2.7	−0.6, 0.6	12.8±2.7	13.5±4.6	−0.8, 0.4
Suprailiac (mm)	8.3±3.0	8.9±3.0	−0.9, 0.4	8.9 ±3.8	9.5±3.9	−0.7, 0.5
Abdomen (mm)	11.4±4.5	12.6±2.9	−1.3, 0.2	11.4±2.9	13.3±5.9	−1.9, 0.1
Thigh (mm)	10.7±3.8	11.4±2.3	−0.9, 0.4	10.0±2.5	11.0±4.8	−1.2, 0.2
Calf (mm)	6.5±1.6	7.7±1.2	−3.7, <0.01*	5.5±1.0	6.1±2.1	−1.7, 0.1
Sum of 7 skinfolds (mm)	63.4±17.1	68.4±10.5	−1.4, 0.2	64.7±15.6	69.8±24.4	−1.5, 0.3

**Physical fitness measure**						

10m speed (s)	2.2±0.1	2.2±0.2	−1.4, 0.2	N/A	N/A	N/A
20m speed (s)	3.5±0.2	3.6±0.2	−0.9, 0.4	3.3±0.2	3.4±0.2	−2.5, 0.01*
40m speed (s)	6.1±0.5	6.2±0.6	−0.5, 0.6	5.6±0.3	5.8±0.4	−3.2, <0.01*
L-run test (s)	6.5±0.3	6.6±0.5	−1.4, 0.2	6.2±0.3	6.3±0.3	−1.7, 0.1
Vertical jump test (cm)^ES^	38.3±2.4	34.9±2.8	6.0, <0.01*	47.8±3.8	42.5±3.8	6.5, <0.01*
Sit and reach (cm)	6.1±5.1	5.1±4.6	1.0, 0.4	N/A	N/A	N/A
2kg MBCT (m)^ES^	7.0±0.7	5.9±0.9	6.0, <0.01*	9.2±1.3	8.3±12	3.5, <0.01*
60s Push Up (n)	38±10	36±9	1.2, 0.2	50±10	44±12	2.4, 0.02*
Wall Sit Leg Strength (s)^ES^	132±7	123±13	3.7, <0.01*	146±10	138±22	2.3, 0.02*
Yo-Yo IRT (m)^ES^	1 307±229	1 030±270	4.7, <0.01*	1 506±76	1 444±259	1.5, 0.1
1 RM BS (kg)	N/A	N/A	N/A	98±15	90±16	2.7, <0.01*
Relative BS (kg/kg^−1^)^ES^	N/A	N/A	N/A	1.3±0.0	1.2±0.1	9.0, <0.01*
1 RM BP (kg)	N/A	N/A	N/A	91±16	81±16	2.9, <0.01*
Relative BP ((kg/kg^−1^)^ES^	N/A	N/A	N/A	1.2±0.1	1.1±0.1	6.6, <0.01*
RHIE total sprint time (s)^ES^	N/A	N/A	N/A	39.3±3.0	41.9±3.0	−4.1, <0.01*

**Rugby-specific game skills**						

Tackling ability test (%)^ES^	83±9	68±8	7.2, <0.01*	88± 8	85±8	2.8, <0.01*
Passing ability test (au)^ES^	106±5	105±4	1.1, 0.3	116±2	113±4	4.5, <0.01*
Catching ability test (au)^ES^	72±2	68±3	6.2, <0.01*	74 ±1	74±1	1.9, 0.1

Playing experience expressed the number of years the players having been playing schoolboy rugby since they started secondary school. A full description of the tackling, catching and passing tests are outlined in the test battery added as a supplementary file.

* significant p-value (p≤0.05);

N/A, not assessed either because of poor reliability coefficients in the test-retest reliability study or other considerations such as relevance and age appropriateness;

ES large effect size.

Df, degrees of freedom; p, probability value for the student independent t-test; 2kg MBCT, 2kg medicine ball chest throw; WSLS, wall sit leg strength; Yo-Yo IR, yo-yo intermittent recovery test level 1; 1RM BS, one repetition maximum back squat; 1RM BP, one repetition maximum bench press; BS, back squat; BP, bench press; RHIE, repeated high intensity exercise test; au, arbitrary units; SD, standard deviation.

**Table 2:** Comparative analysis of physical characteristics and rugby-specific game skills between U16 Zimbabwean schoolboy rugby players and others (Mean±SD)

Variables	Elite U16 Zimbabwe (1)	Elite U16 SA (2)	Elite U16 England (3)	Elite U16 NZ (4)	Elite U16 France (5)	Effect size

Chronological age (y)	14.9±0.3	U16*	15.5±0.30^b^	15.4±1.40^d^	U16*	2.0 (1) vs (3)
Playing experience (y)	2.5±0.5	-	-	-	-	-

**Anthropometrics**						

Body mass (kg)	63.7±9.1	76.2±11.7^c^	79.4±12.8^b^	79.7±15.2^d^	84.0±9.1	2.2 (1) vs (5)
Height (m)	1.7±0.1	1.8±8.1^c^	1.8±7.1^b^	1.8±3.9^d^	1.8±6.8	0.0 (1) vs (5)
Sum of 7 skinfolds (mm)	63.4±17.1	67.6±28.3^c^	-	-	-	0.2 (1) vs (2)

**Physical fitness measure**						

10m speed (s)	2.2±0.1	1.9±0.1^c^	1.8±0.1^b^	1.8±5.4^d^	1.7±0.1	4.0 (1) vs (5)
20m speed (s)	3.5±0.2	-	3.1±0.2^b^	3.1±6.0^d^	-	2.0 (1) vs (3)
40m speed (s)	6.1±0.5	5.5±0.2^c^	5.7±0.4^b^	-	-	1.7 (1) vs (2)
L-run test (s)	6.5±0.3	-	-		-	-
Vertical jump test (cm)*	38.3±2.4	47.2±6.1^e^	-	50.1±7.0^e^	-	2.3 (1) vs (4)
Sit and reach (cm)	6.1±5.1	2.4±2.3^e^	-	−2.2±8.8^e^	-	1.0 (1) vs (2)
2kg MBCT (m)*	7.0±0.6	-	-	-	-	-
60s Push Up (n)	38 ±10	39±11^c^	-	-	-	0.0 (1) vs (2)
Wall Sit Leg Strength (s)*	132±7	-	-	-	-	-
Yo-Yo IRT (m)*	1 307±229	-	1 144±337^b^	-	-	0.6 (1) vs (3)

**Rugby-specific game skills**						

Tackling ability test (%)	83±9	-	-	-	-	-
Passing ability test (au)	106±5	-	-	-	-	-
Catching ability test (au)	72±2	-	-	-	-	-

* not categorically stated in the article. French players average values for forward and backline players were computed (n=89) as reported Peeters et al. [17] study.

b , Darral Jones et al. [16];

c , Spamer and De la Port [19];

d , Smart and Gill [18];

e , Spamer et al. [10],

2kg MBCT, 2kg medicine ball chest throw; Yo-Yo IR level, yo-yo intermittent recovery level 1 test; SD, standard deviation; SA, South Africa; Zim, Zimbabwe.

**Table 3:** Comparative analysis of physical characteristics and rugby-specific game skills between U16 Zimbabwean schoolboy rugby players and others (Mean±SD)

Variables	Elite U16 Zimbabwe (1)	Elite U16 SA (2)	Elite U16 England (3)	Elite U16 France (5)	Effect size

Chronological age (y)	17.5±0.9	17.4±0.70^b^	17.5±0.6^a^	U18^*^	0.1 (1) vs (2)
Playing experience (y)	2.5±0.5	-	-	-	-

**Anthropometrics**					

Body mass (kg)	63.7±9.1	79.3±10.5^b^	78.4±12.9^a^	92.6±9.1	3.2 (1) vs (4)
Height (m)	1.7±0.1	1.8±6.9^b^	1.8±10.0^a^	1.84±6.4	0.0 (1) vs (4)
Sum of 7 skinfolds (mm)	63.4±17.1	60.3±20.9^b^	-	-	0.2 (1) vs (2)

**Physical fitness measure**					

10m speed (s)	2.2±0.1	1.9±0.1^c^	1.8±0.1^a^	1.72±0.1	4.3 (1) vs (4)
20m speed (s)	3.5±0.2	-	3.2±0.2^a^	-	1.7 (1) vs (3)
40m speed (s)	6.1±0.5	5.4±0.3^c^	6.0±0.3^a^	-	1.8 (1) vs (2)
L-run test (s)	6.5±0.3	-	-	-	-
Vertical jump test (cm)	38.3±2.4	-	-	-	-
Sit and reach (cm)	6.1±5.1	-	-	-	-
2kg MBCT (m)^*^	7.0±0.6	-	-	-	-
60s Push Up (n)	38±10	51±27^c^	-	-	0.6 (1) vs (2)
Wall Sit Leg Strength (s)	132±7	-	-	-	-
Yo-Yo IRT (m)	1 506±76	-	1 022±515^a^	-	1.3(1) vs (3)
Relative BS (kg/kg-1)	1.3±0.0	-	-	-	-
1 RM BP (kg)	91±16	95±19^c^	-	106±15	1.0 (1) vs (2)
Relative BP ((kg/kg-1)	1.2±0.1	-	-	-	-
RHIE total sprint time (s)	39±3	-	-	-	-

**Rugby-specific game skills**					

Tackling ability test (%)	88±8	-	-	-	-
Passing ability test (au)	116±2	-	-	-	-
Catching ability test (au)	74±1	-	-	-	-

a , Jones et al. [12];

b -de ridder et al [20];

c , Spamer and Delaport for U18 SA players [19],

2kg MBCT, 2kg medicine ball chest throw; WSLS, wall sit leg strength; Yo-Yo IR, yo-yo intermittent recovery test; 1RM BS, one repetition maximum back squat; 1RM BP, one repetition maximum bench press; BS, back quat; BP, bench press; RHIE, repeated high intensity exercise test; au, arbitrary units.

## Data Availability

All relevant data is included in the paper, along with supporting information and supplementary files.
