# Supplementary material for: A comparative analysis of physical fitness characteristics and rugby-specific game skills of schoolboy players by playing standards: Implications for talent identification programs in resource-constrained settings
Source: S Afr J Sports Med. 2024 Jan 15;36(1):v36i1a18525. doi: 10.17159/2078-516X/2024/v36i1a18525 (PMC11629942; doi:10.17159/2078-516X/2024/v36i1a18525)
Supplement: Supplementary file 1 [file 2078-516X-36-v36i1a18525-s001.pdf]

A comparative analysis of physical fitness characteristics and rugby-specific game skills of schoolboy players by playing standards: Implications for talent identification programs in resource-constrained settings

1. **Height/Stature (cm)**

Height was measured using a portable stadiometer (Seca Model 213, Hamburg Germany). The procedure involved lowering the stadiometer to participant's head and recording the height to the nearest 0.1cm. The participants were measured twice and the mean was recorded.

2. **Body mass (kg)**

Body mass was measured to the nearest 0.1kg using a calibrated digital flat scale. The participants were measured twice and the mean was recorded.

3. **Skinfold measurements (mm)**

The sum of seven site skinfolds was determined using calibrated Lange skinfold calipers. The general procedure involved gently and firmly "pinching" the skin and the subcutaneous fat between the thumb, forefinger and middle finger. The researcher opened the skinfold caliper and measured the skinfold approximately 1cm below the finger and 1cm deep into the skinfold. Two measurements per site were measured and had to agree within 1 millimeter.

4. **Speed tests (5m, 10m, 20m, 40m)**

The participants ran the 5m distance first, followed by 10m, 20m and 40m speed test. Participants started from a stationary position. The players set off in their own time and run maximally through the specified distance. Two test trials were run for each distance and the better of the two was recorded.

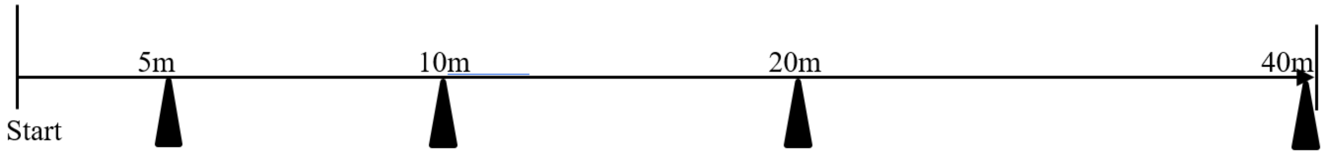

Fig.1. *Schematic representation of speed test*

5. **L-run agility test (sec)**

Three cones were placed 5m apart from each other to represent an 'L' shape. Players started 1m from the first cone lying in a prone position. At the first cone, a rugby ball was placed and participants were instructed to get up on "GO" command and pick up the rugby ball and run quickly along the 5m, turn left, run forward in between the cones, turn 180 degrees, run straight to finish and score a "try" or drop the ball at the 1m line. Hand-held stopwatches recorded the time. Two trials were done and the best count was recorded.

6. **Vertical jump (VJ) test (cm)**

Players stood with fleet flat, shoulder width with the wall sideward to dominant side. Participants extended arm and hand and mark the highest point with the chalk. The player went to a crouched position, close to 90 degrees of knee flexion keeping the feet flat. They would hold a new full-length chalk between the tips of the fingers every time and spring upward as high as possible and mark the highest point on the wall. The research assistant drew a small line with a ruler on top of the dot made by the chalk representing the maximum height reached. Two trials were permitted. The best count was recorded in cm using a tape measure.

7. **Sit-and-Reach (SR) test (cm)**

Participants sat on the ground, knees extended, and soles of the feet contacting the rigid sit-and-reach box. Each participant would stretch as far as possible with stacked hands and hold that position for one second. Each player was allowed 3 maximum practice attempts before test trials. During test trials, two attempts were made and the best score recorded.

8. **60 seconds push-up test**

Players began in prone, hands on the floor, thumbs and shoulder width apart and elbows fully extended. Players descended to the tester fist placed on the ground below the players' sternum and then ascend until elbows are straight. Push-up action was to be

continuous with a single rest of no more than 2 seconds permitted between repetitions. Athletes performed the maximum number of push-ups in 60 seconds. If the athlete fails to complete the full 60 seconds due to fatigue, this failure was recorded together with the number full repetitions recorded and the time of drop-put

9. 2kg medicine ball chest throw (2kg MBCT)

Players threw a 2kg medicine ball horizontally as far as possible while seated with the back, and legs straight. Distance was measured using the tape measure to the nearest 0.1m from the sitting line to where the ball initially landed with the higher of two trials measured.

10. Wall sit leg strength test (WSLS) (seconds)

Participants stood comfortably with back against a vertical wall. Participants slided the back down maintaining contact with the wall to assume a position with both knees and hip of 90° angle. However, the timing started when one foot was lifted off the ground and was stopped when the subject could not maintain that position and the foot is returned to the ground. The non-dominant leg was lifted to measure the leg strength of the dominant side.

11. Repeated high intensity exercise test (RHIE) (seconds)

The RHIE test was devised in two parts.

Back test

For backline players, each player completed three 20-m sprints before decelerating and jogging to the start (Fig 2). The sprints were performed on a 20-second turnaround, with approximately 16–17 second active recovery. The players had to sprint with maximal effort. After completing 3 sprints, the player moved to the side of the running lane and completed 2 tackles. They sprinted 10m to the tackle a bag, driving it for 2 m. They then ran backward to the start and 20 seconds later completed a second tackle. A 20-second recovery followed the tackle shuttle; the player then repeated the 3 by 20-m sprint protocol and tackles drill.

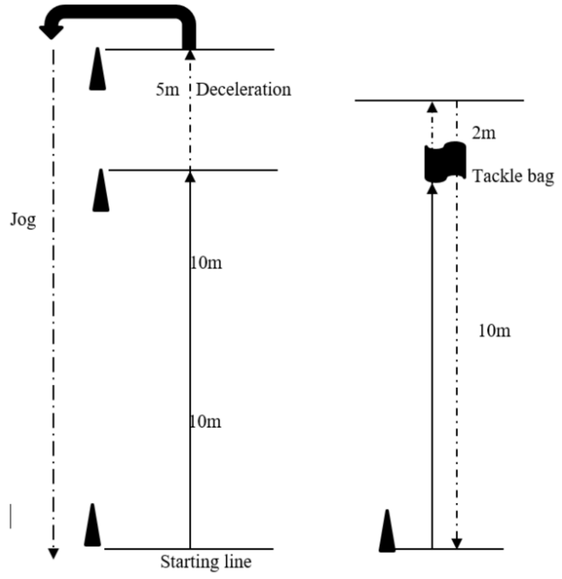

Fig. 2. *Schematic of backline players RHIE test*

Forward test

Each player completed 3 by 20-m sprints before decelerating and jogging to the start (Fig 3). Each sprint was performed on a 20-second cycle, leaving 16- to 17-second recovery between sprint efforts. After the third sprint in each set and after a 60-second rest, the player completed a “scrum sled shuttle”. This involved pushing a weighted (50 kg) one-man scrum sled for 5 m in 1 direction and 5 m back to the start. The player completed these 4 times, with a 10-second rest between bouts. On completion, the player was given 20 seconds to return to the sprint lane. Participants repeated the sprint shuttles before moving to the tackle drill. After a 60-second rest, they sprinted 10 m and tackled a tackle bag driving it at least 2m. On completion of the tackle, players ran backward to the start line and repeated the tackle drill on 4 occasions; 20 seconds separated the start of each sprint to tackle. Times were recorded for each sprint repetition (total of 9).

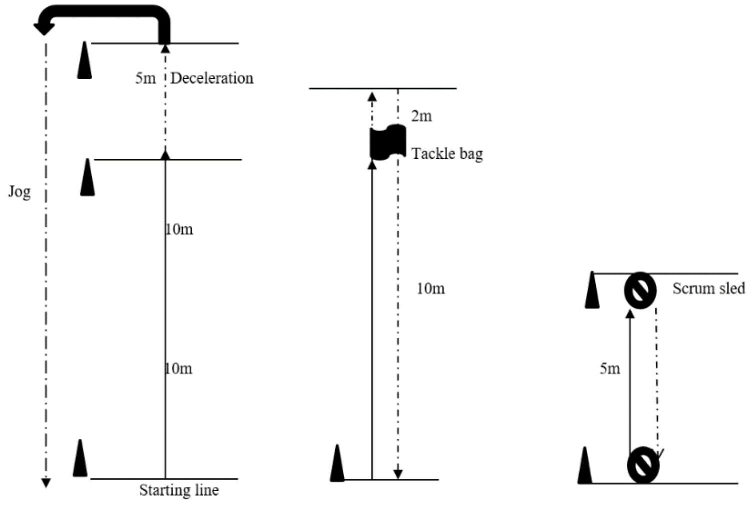

Fig. 3. *Schematic of forward players RHIE test*

Total RHIE performance sprint time was taken as the sum of all three sets of sprints (nine 20-m sprints in total). Decrement in sprint performance was calculated as the difference in time taken (seconds) to complete the third set of sprints (sprints 7–9) compared with the total time taken to complete the first set of 3 sprints (sprints 1–3).

12. Yo-Yo intermittent recovery test (m)

Starting from a stationary position, participants run back and forth along the 20m track while keeping in time with the audio signals played from the compact disk. The players started after the beep and runs to opposite line. The players turned when the next beep goes. After about 1 minute, a sound indicated an increase in speed, and the beeps got closer together with time. If the line was reached before the beep, the subjects waited until the sound beeps before continuing. If the line was not reached before the beep, the participant was given a warning and had to continue running to the line, then turn and try to catch up with the pace within two more beeps. We recorded the number of complete laps run and shuttles. This was used to calculate the total Yo-Yo distance in meters.

13. One repetition maximum back squat (kg)

Participants had to lower the barbell to a depth equivalent to at least 90 of knee flexion. The greater trochanter of the femur had to be aligned with the patella and on ascension the knee and hip had to be in full extension. If the set was successfully completed then weight was added and if not weight was reduced and another set attempted. A 3-5 minutes rest was provided between each set. This process of adding and removing was continued until a 1RM was achieved. The players' 1RM scores were divided by the body mass to provide a strength score relative to body mass.

14. One repetition maximum bench press (kg)

Participants lowered the barbell to touch the chest and pushed the barbell until the elbows were locked out. Participants used a self-selected hand position and were required to lower the bar at approximately 90-degree angle at the elbows and then pressed the bar in a vertical position so that the arms are fully extended.

15. Tackling proficiency test

A simulated rugby-specific match 2-on-1 scenario was simulated within a 10×10 m grid for the tackling test. A local rugby coach with experience served as an expert rater (obs 1) for the skill rating. Three players were used at one given time. The two attacking players were instructed to advance from one side of the grid to the other and complete **one pass each** before being tackled by the defending player (test player). The attacking players had to make passes between them within the 3m while advancing forward. The procedure was for the test player to tackle the player with the ball. Six test trials were conducted for observer to observe the tackling skills under fatigue.

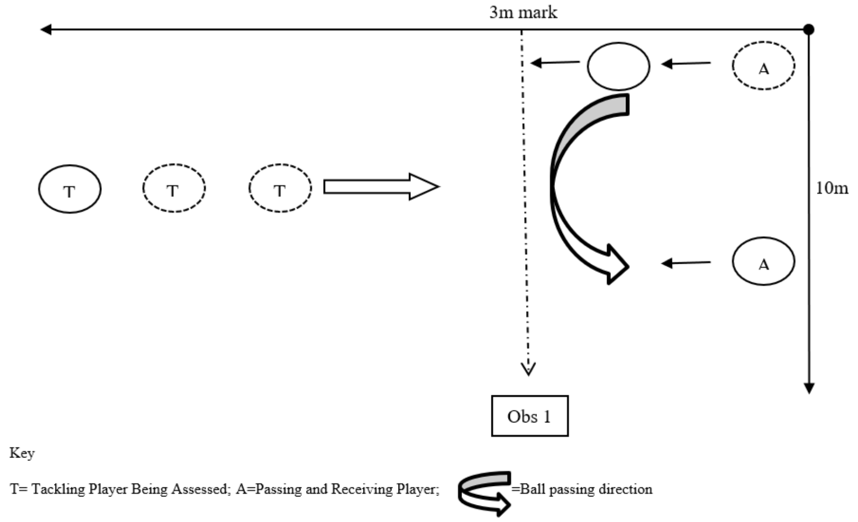

Fig. 4. *Illustration of the tackling protocol*

The observer assessed and scored each of those six trials based on technical checklist. The observer rated each player in real time on their overall proficiency in each skill using a Likert scale (*0-not achieved completely; 1-partly achieved; 2-completely achieved relevant criteria)*. The technical criteria were as follows:

1. *Contacting the target in the centre of gravity/low body position*
2. *Contacting the target with at least one shoulder*
3. *Head to one side of the body*
4. *Arms should readiness for tackling*
5. *Body position square/aligned*
6. *Arms completely wrapped around the target on contact when tackling*
7. *Leg drive on contact/ drive with the legs*
8. *Centre of gravity forward on base of support*
9. *Maintain grip until the attacking player is on ground*
10. *Turtle the player/ hold the player immobile on the ground/defensive shape*

16. Passing-for-accuracy over 7m test and passing ability skill test

This test was used for combined assessment of passing skill ability and accuracy in passing for over a 7m distance. Participants commenced chest down and knees extended behind the starting line. They stood up on the word "go" and grabbed a rugby ball on the touch line and sprint in a zig-zag way on a 10m course. Upon entering the passing grid zone, they released the rugby ball and prepared to receive a pass from one expert coaches (E1). There were instructed to catch the ball and pass immediately at a moving target (R) placed at a 7m distance with a defensive player approaching to offer a hindrance. Rugby expert (E2) rated the pass made by the first expert (E1). After each pass, the subject ran back to the starting line and repeated the test. Overall, the total passes made were 15 executed in three sets of 5. The number of accurate passes made (passes caught) to the receiver (R) was determined by the lead author observing and expressed as percentage of the total passes made to give the passing accuracy (%) score for the participant. In addition, an expert rugby coach (E2) judged passing ability looking at the eight elements giving a passing ability score for each pass. The scoring was based on a dichotomous response scale: *0-not achieved; 1-achieved*. So each participant was assessed 15 times (3 sets of 5) and a score was recorded for each technical element. All the scores were then added for each test trial to give a total passing skill score reflecting passing ability score in arbitrary units. The technical criteria assessed:

1. *Pendulum action*
2. *Looking where pass is to be made*
3. *Single movement*
4. *Straight follow through of passing hand*
5. *Appropriate ball speed*
6. *Pass in front of the receiver*
7. *Receiver catches the ball*
8. *Receiver maintain stride/minimal breaking of the receiver pace to receive the ball*

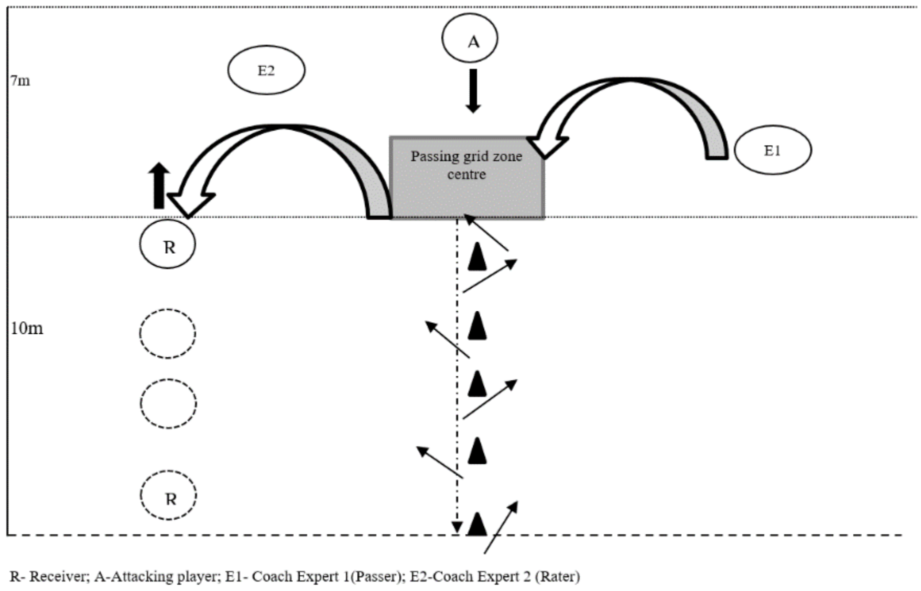

Fig. 5. *Passing protocol for passing ability and accuracy assessment*

17. Running-and-catching ability test

The protocol was similar to the passing protocol and was mainly designed for the assessment of catching ability skills test based on expert rating. Participants ran in a zigzag fashion for 10m holding the rugby ball. The starting position of the prone was similar to the passing procedure. Upon entering the catching grid zone (measured 3m by 3m), the ball is passed immediately from 7m, and the test player should show ability to catch the ball. The test was performed on a different day from the passing protocol to allow for independent assessment of the skills accurately.

The technical criteria looked at the following elements:

1. *Eyes on the ball/Focus on passer/ Body receptive to the pass*
2. *Hands up/elbows bent/*
3. *Fingers spread/palms out and thumbs up*
4. *Take the pass early/meet the ball early*
5. *Catch the pass/Hold the "body" of the ball and all this with minimal breaking of the natural or starting stride of the player*.

Each of the five criteria was assessed based on a Likert scale from 0 (failed completely to perform the activity), 1 (completely achieved). Participants were assessed 15 times (3 sets of 5). The idea is to see how the participants would fare before and after fatigue has set in. The total score per test trial was 5 aggregating to 75 after completion of 15 tests trial
